# *In vitro* screening and characterization of lactic acid bacteria from Lithuanian fermented food with potential probiotic properties

**DOI:** 10.3389/fmicb.2023.1213370

**Published:** 2023-09-08

**Authors:** Ashwinipriyadarshini Megur, Eric Banan-Mwine Daliri, Toma Balnionytė, Jonita Stankevičiūtė, Eglė Lastauskienė, Aurelijus Burokas

**Affiliations:** ^1^Department of Biological Models, Institute of Biochemistry, Life Sciences Center, Vilnius University, Vilnius, Lithuania; ^2^Department of Molecular Microbiology and Biotechnology, Institute of Biochemistry, Life Sciences Center, Vilnius University, Vilnius, Lithuania; ^3^Department of Microbiology and Biotechnology, Institute of Biosciences, Life Science Center, Vilnius University, Vilnius, Lithuania

**Keywords:** fermented food, lactic acid bacteria, potential probiotics, *in vitro* screening, tryptophan, safety assessment, probiotics LAB-lactic acid bacteria, GIT-gastrointestinal tract

## Abstract

The present work aimed to identify probiotic candidates from Lithuanian homemade fermented food samples. A total of 23 lactic acid bacteria were isolated from different fermented food samples. Among these, only 12 showed resistance to low pH, tolerance to pepsin, bile salts, and pancreatin. The 12 strains also exhibited antimicrobial activity against *Staphylococcus aureus* ATCC 29213, *Salmonella Typhimurium* ATCC 14028, *Streptococcus pyogenes* ATCC 12384, *Streptococcus pyogenes* ATCC 19615, and *Klebsiella pneumoniae* ATCC 13883. Cell-free supernatants of isolate 3A and 55w showed the strongest antioxidant activity of 26.37 μg/mL and 26.06 μg/mL, respectively. Isolate 11w exhibited the strongest auto-aggregation ability of 79.96% as well as the strongest adhesion to HCT116 colon cells (25.671 ± 0.43%). The selected strains were tested for their synbiotic relation in the presence of a prebiotic. The selected candidates showed high proliferation in the presence of 4% as compared to 2% galactooligosaccharides. Among the strains tested for tryptophan production ability, isolate 11w produced the highest L-tryptophan levels of 16.63 ± 2.25 μm, exhibiting psychobiotic ability in the presence of a prebiotic. The safety of these strains was studied by ascertaining their antibiotic susceptibility, mucin degradation, gelatin hydrolysis, and hemolytic activity. In all, isolates 40C and 11w demonstrated the most desirable probiotic potentials and were identified by 16S RNA and later confirmed by whole genome sequencing as *Lacticaseibacillus paracasei* 11w, and *Lactiplantibacillus plantarum* 40C: following with the harboring plasmid investigation. Out of all the 23 selected strains, only *Lacticaseibacillus paracasei* 11w showed the potential and desirable probiotic properties.

## Introduction

The demand for functional foods has increased in recent years due to consumers’ interest in their therapeutic applications. The main types of functional foods include probiotics, prebiotics, and synbiotics (which are a mixture of probiotics and prebiotics) (Topolska et al., 2021). The demand for different strains of probiotics has led to consumer awareness of the health benefits and therapeutic effects of modulating the gut microbiota, leading to the amelioration of neurological diseases and metabolic disorders (Amoah et al., 2022). These health benefits are due to the direct effects of the microbes on the host and their fermentation products in the gut. Because these fermented by-products of microbes remain in the gut after consumption, they play a significant role in the functioning of the body and may have direct health benefits for the host.

The Food and Agriculture Organization and the World Health Organization define probiotics as live microorganisms that confer health benefits on their hosts when ingested in an adequate concentration (Salminen et al., 2021). Over the last decades, studies on probiotics have expanded tremendously. Numerous *in vivo* studies have found that, when adequately administered, probiotics modulate the gut microbiota by promoting the growth of beneficial microorganisms in the gastrointestinal tract (GIT) (Vadopalas et al., 2020).

Most strains of lactic acid bacteria (LAB) are commonly used as probiotics in foods (Zapaśnik et al., 2022). LAB are a group of bacteria that include genera such as *Lactobacillus*, *Lactococcus*, *Pediococcus*, *Enterococcus*, and *Streptococcus*, are Gram-positive cocci or rods, and are acid-tolerant, non-respiring but aerotolerant bacteria (Shokryazdan et al., 2017). They are naturally present in fermented foods, composts (Tran et al., 2019), GIT (Marchwińska and Gwiazdowska, 2021), vaginal tract (Silva et al., 2022), plant surfaces (Yu et al., 2020), and silages (Bohn et al., 2017).

Probiotic fermentation often produces by-products with diverse health-promoting effects, including protection against infectious agents (Bartkiene et al., 2020; Mileriene et al., 2023), anti-allergenic effects (Liang et al., 2022), immunomodulatory effects (Kober et al., 2022), anti-obesity effects (Liu et al., 2022), antidiabetic effects (Wang et al., 2022; Daliri et al., 2023a), antioxidant effects (Hoffmann et al., 2021), enhancement of the bioavailability of vitamins/minerals (Ballini et al., 2019), anti-anxiety effects (Lalonde and Strazielle, 2022), and attenuation of Alzheimer’s disease (Megur et al., 2021).

In recent years, many probiotic candidates have been isolated from traditionally fermented foods and their potential effects on health have been well documented. For instance, *Pediococcus acidilactici* SDL 1402 and *Weissella cibaria* SCCB 2306 isolated from Korean fermented soybean paste were shown to survive simulated gastrointestinal conditions, inhibit pathogenic bacteria, and showed good gut colonization potentials (Oh et al., 2018). The bacteria were found to have no virulent factors and displayed significant cholesterol-reducing potentials *in vivo* (Daliri et al., 2022). Similarly, *Lacticaseibacillus paracasei* L2 isolated from Lben (a Tunisian traditionally fermented dairy product) displayed an excellent gut colonization potential, strong pathogen inhibiting ability, and produced antioxidant metabolites during fermentation (M’hamed et al., 2023). Traditionally fermented foods, therefore, remain a good source of probiotic candidates since most of their commensal LAB are generally regarded as safe or qualified presumption of safety (Koutsoumanis et al., 2021; Grujović et al., 2022).

The criteria for the selection of probiotic strains are considered important before their use in animal and/or human studies. The most important feature of a probiotic is its potential health effect and safety. Desirable properties of probiotics include their ability to survive in the GIT, their antimicrobial activity against pathogenic microorganisms, and their antioxidant properties (Reuben et al., 2020). Furthermore, the binding ability of LAB to HCT116 colon cells has been used as a criterion for assessing the potential gut colonization ability of probiotic candidates in some studies (M’hamed et al., 2023). In addition, their ability to grow in the presence of prebiotics to produce essential metabolites such as tryptophan are considered desirable traits (Kepert et al., 2017). For this reason, the present study aimed to isolate probiotic bacteria from Lithuanian fermented pear, cherry tomato, cucumber, and orange. The isolates were screened for their resistance to simulated gastrointestinal conditions. Strains that survived *in-vitro* gastrointestinal conditions were tested for their functional properties and the selected strains were tested for their safety. Probiotic candidates were identified using whole genome sequencing.

## Materials and methods

### Sample collection, bacteria isolation, and selection

Strains were isolated from various fermented foods collected at Halės Turgus market (Halle Market, Vilnius, Lithuania). The fermented foods were traditional Lithuanian fermented cherry tomatoes, pears, oranges, and cucumbers. Each sample paste (1 g) was transferred aseptically into separate test tubes containing 9 mL of sterile peptone water (0.1%) (Sigma- Aldrich, Poznań, Poland). Aliquots of 10 μL from appropriate 10^5^ CFU/mL dilution were pentagonally streaked on the pre-solidified de Man, Rogosa, and Sharpe (MRS) agar (Oxoid, Wesel, Germany) and incubated at 37°C for 48–72 h under aerobic conditions. Representative colonies of LAB were randomly picked and were purified by repeated streak plating on MRS agar until pure colonies were obtained. The pure colonies were maintained on MRS agar plates and subcultured every 5 weeks until imperative for characterization. Cell morphology and colonial characterization were observed on MRS agar. These isolates were stored and preserved in a −80°C deep freezer (Froilabo, Livingston, United Kingdom) at the Department of Microbiology, Faculty of Life Sciences Centre, Vilnius University, Vilnius, Lithuania.

### Cell culture

The human colonic cell lines HCT-116 were obtained from the Department of Biological Models, Vilnius University, Lithuania. The cells were routinely cultured in Dulbecco’s modified Eagle’s minimal essential medium (DMEM; Sigma- Aldrich, Poznań, Poland) supplemented with 10% (v/v) heat-inactivated (30 min, 56°C) fetal bovine serum (Sigma- Aldrich, Poznań, Poland). The cells in a medium were also supplemented with a 1% (v/v) penicillin–streptomycin solution to a final concentration of 100 U mL^−1^ penicillin and 100 μg mL^−1^ streptomycin. The incubation was at 37°C in an atmosphere of 5% CO_2_ and 95% air. The cells were nourished with complete DMEM every alternate day until the cells reached 70–80% confluency.

### Screening of probiotic properties of isolated bacteria

#### Resistance to low pH

The resistance of LAB to low pH was studied according to a previously described method (Oh et al., 2018), with little modifications. Briefly, LAB cultures incubated at 37°C for 24 h were centrifuged at 10,000 g for 10 min. The pellets were suspended in sterile PBS (Sigma- Aldrich, Poznań, Poland) and adjusted to a pH of 2.0 using 1 M HCl. The mixture was then incubated at 37°C for 4 h. Aliquots of the mixture were taken at time 0 and after 4 h. The samples were serially diluted in peptone water and the viable cells were determined by the spread plate method using MRS agar. The plates were incubated at 37°C for 24 h and the percentage survival of the bacteria was calculated as follows:


$$\%\;\mathrm{Survival}=\left(\frac{CFU*\mathrm{of viable cells survived}}{CFU*\mathrm{of initial viable cells inoculated}}\right)\times 100$$

*CFU = Colony forming units.

#### Resistance to pepsin

To test the viability in the presence of pepsin, simulated gastric juice was prepared by suspending 3 mg/mL pepsin (Sigma- Aldrich, Poznań, Poland) in sterile peptone water (w/v) and adjusted to pH 2.0. The fluid was inoculated with active cultures at an inoculum size of 1% (v/v) and incubated at 37°C for 4 h. The viable cells were determined before (T1) and after incubation (T2) by the spread plate method (Tokatl et al., 2015). The percentage survival of the bacteria was calculated according to resistance to low pH.


$$\%\;\mathrm{Survival}=\left(\frac{CFU*\mathrm{of viable cells survived}}{CFU*\mathrm{of initial viable cells inoculated}}\right)\times 100$$

*CFU = Colony forming units.

#### Resistance to bile salts and pancreatin

Resistance to intestinal juices was tested as reported (Manovina et al., 2022). Briefly, 0.3% (w/v) bile salt (Sigma- Aldrich, Poznań, Poland) and 1 mg/mL pancreatin (Sigma- Aldrich, Poznań, Poland) were dissolved in sterile peptone water (w/v) adjusted to pH 8 cell-free culture supernatants. The fluid was inoculated with 1% (v/v) LAB cultures and incubated at 37°C for 6 h. The viable cells were determined before and after incubation by the spread plate method. The percentage survival of the bacteria was calculated according to the equation below.


$$\%\;\mathrm{Survival}=\left(\;\frac{CFU*\mathrm{of viable cells survived}}{CFU*\mathrm{of initial viable cells inoculated}}\right)\times 100$$

*CFU = Colony forming units.

### Assessment of functional properties

#### Probiotic antimicrobial activity

Antibacterial activity was determined using the agar well diffusion test as previously described (Edith Marius et al., 2018). *Staphylococcus aureus* ATCC 29213, *Salmonella Typhimurium* ATCC 14028, *Streptococcus pyogenes* ATCC 12384, *Streptococcus pyogenes* ATCC 19615, and *Klebsiella pneumoniae* ATCC 13883 were obtained from the Department of Microbiology, Vilnius University and were used as indicator strains for the detection of antimicrobial activity. The LAB were cultured in 3 mL MRS broth medium and incubated for 24 h at 37°C. The MRS broth tubes were subsequently centrifuged (10000 rpm for 10 min) to prepare cell-free culture supernatants (CFS). The pH values of the supernatants were adjusted to approximately 7 by the addition of NaOH. A suspension of 100 μL of 10^7^ CFU/mL of each pathogenic strain was then prepared and spread onto the nutrient agar, into which 5-mm-deep wells had been dug. Approximately 100 μL of CFS was poured into each well, and nutrient agar plates were incubated for 24 h at 37°C. Finally, the inhibition zone diameter was measured in millimeters (mm) (Xu et al., 2020).

#### Trolox equivalent antioxidant capacity

The scavenging effect of the CFS on a 1,1-Diphenyl2-picryl-hydrazyl (DPPH) radical was assessed as described in a previous study (Liao et al., 2012). Briefly, the scavenging ratio of the sample and Trolox (Abcam, Cambridge, United Kingdom) on DPPH (Abcam, Cambridge, UK) at the same time was tested, and then a suitable concentration range of the Trolox and its scavenging percentage was found. Then, a linear regression equation between the Trolox concentration and its scavenging percentage was built, and the Trolox equivalent antioxidant capacity (TEAC) was calculated through the equation. A higher TEAC value meant higher DPPH scavenging activity. Meanwhile, the scavenging percentage on the DPPH radical of the sample solution was tested following the treatment of the Trolox solution. The scavenging effect on the DPPH radical of the samples could be calculated as the Trolox equivalent’s antioxidant capacity from the calibration curve: y=−0.0298x+0.9995.

### *In vitro* gut colonization potential

Auto-aggregation ability was determined by the method described previously with slight modification (Botta et al., 2014; Li et al., 2020). The overnight selected LAB culture was centrifuged at 10000 rpm for 10 min to harvest the cell pellets. Pellets were washed thrice with phosphate-buffered saline (PBS; pH 7.4), re-suspended in PBS, and the initial absorbance was noted at 600 nm. The bacterial suspension was incubated at 37°C for 24 h, and the final absorbance of the supernatant was measured at 600 nm at three different times: 4 h, 12 h, and 24 h. The percentage of cellular auto-aggregation was measured by the formula:


$$\%\;\mathrm{Auto-aggregation}=\left(\;\frac{\mathrm{ODinitial}-\mathrm{ODfinal}\;}{\mathrm{ODinitial}}\right)\times 100$$

### *In vitro* percent adhesion on HCT116 cells

The concentration of cells in a monolayer was determined by trypsinizing the adhered cells with 3 mL of 0.25% trypsin– EDTA solution for 5–10 min at 37°C. The final cell count in suspension was measured with the help of a hemocytometer (Sigma- Aldrich, Poznań, Poland). For adhesion assay, HCT116 cells were seeded separately in each well of standard 12-well tissue culture plates at a concentration of 1×10^6^ cells/mL and incubated for ~48 h or more, until a complete monolayer was obtained. Change of medium was performed every 24–48 h. The spent medium was completely removed 24 h before adhesion assay and cells were fed with DMEM lacking antibiotics. The LAB isolates for adhesion assay were propagated in MRS broth and cultures obtained after 18 h of growth at 37°C were centrifuged at 6000 × g for 10 min. The pellet was washed once with PBS (pH 7.4). The cell density was adjusted approximately to the desired levels by measuring the absorbance at 600 nm. The exact number of viable bacteria used in the assay was determined by plate counting on MRS agar.

The adhesion of LAB isolates was measured as described previously with few modifications (Sharma and Kanwar, 2017). The HCT 116 cells in a monolayer were washed twice with 3 mL of PBS (pH 7.6). The 2 mL of DMEM without serum and antibiotics was added to each well and incubated at 37°C for 40 min before inoculation of bacteria. Different LAB isolates with a concentration of approximately 1 × 10^7^ CFU suspended in 1 mL DMEM without serum and antibiotics were used to inoculate each well of tissue culture plates. The plates were incubated at 37°C in an atmosphere of 5% CO_2_ and 95% air for 3 h. After incubation, the monolayer was washed five times with sterile PBS (pH 7.6) to remove non-adherent bacteria.

The monolayer was washed five times with sterile PBS (pH 7.4) to remove non-adherent bacteria. To enumerate the viable adhered bacteria, the cells from the monolayer were detached by trypsinization. Each well was treated with 1 mL of 0.25% trypsin–EDTA solution and incubated for 15 min at room temperature. The suspension of lysed cells and LAB was serially diluted with saline solution and plated on MRS agar. The enumeration was done after 48 h of incubation at 37°C in an anaerobic atmosphere. The adhesion was expressed as the percentage of the number of adhered bacteria to the total bacteria used for the experiment and calculated as:


$$\mathrm{Percent adhesion}=\frac{B1}{B0}\times 100$$

where B0 and B1 CFU/mL are the initial and final count of bacteria, respectively.

### Influence of galactooligosaccharides on strain growth

Prebiotic influence on LAB growth was tested as described previously with slight modifications (Wang et al., 2019). Briefly, LAB cultures incubated at 37°C for 24 h were administered in the MRS broth containing prebiotic 2% Galactooligosaccharides (GOS) and 4% GOS, i.e., the lowest concentration that elicited a significant increase in the growth of LAB (data not shown). The LAB growth was noted at every 4 h interval at 37°C by measuring absorbance at 600 nm. The optical densities were measured using a spectrometer (Eppendorf Bio spectrometer®, Hamburg, Germany). The initial optical density value of the media was deducted from the final value for each test sample.

### Tryptophan-producing ability of LAB

The tryptophan production by LAB was monitored as previously described (Vaitekūnas et al., 2020). In the prebiotic-supplemented LAB CFS samples, concentrations of tryptophan were determined by high-performance liquid chromatography-mass spectrometry (HPLC-MS). First, the samples were mixed with an equal volume of acetonitrile and centrifuged for 10 min at 10,000 rpm. The samples were analyzed using the Shimadzu Prominence HPLC system (Shimadzu, Kyoto, Japan) equipped with a photodiode array (PDA) detector (Shimadzu, Kyoto, Japan) and LCMS-2020 mass spectrometer (Shimadzu, Kyoto, Japan) with an electrospray ionization (ESI) source. The chromatographic separation was conducted using a YMC Pack Pro C18 column (3 × 150 mm; YMC, Kyoto, Japan) at 40°C and a mobile phase that consisted of 0.1% formic acid water solution (solvent A) and acetonitrile (solvent B) delivered in the 5–95% gradient elution mode. Mass scans were measured from m/z 50 up to m/z 2,000 at a 350°C interface temperature, 250°C desolvation line (DL) temperature, ±4,500 V interface voltage, and neutral DL/Qarray, using N_2_ as nebulizing and drying gas. Mass spectrometry data were acquired in both positive and negative ionization modes. The data were analyzed using LabSolutions software (Shimadzu, Kyoto, Japan).

### DNA extraction and molecular identification

Genomic DNA was isolated from sediment samples using the ZymoBIOMICS™ DNA Miniprep Kit (Zymo Research, Seattle, United States) according to the manufacturer’s recommendations. The concentration of extracted DNA was evaluated using an Eppendorf bio photometer (Eppendorf, Hamburg, Germany) (Lastauskienė et al., 2021). The molecular identification of LAB strains was conducted by 16S RNA and later confirmed by whole genome sequencing analysis. For 16S RNA sequencing, the strains were sent to Microgen, Netherlands, and for the whole genome sequencing the strains were sent to Cosmos, USA. Each sequence amplicon was BLAST^®^ analyzed and aligned with the National Center for Biotechnology Information (NCBI) Sequence comparison database^1^ to determine the sequence identity and GenBank accession number. A phylogenetic tree was constructed after para-wise alignment applying CLUSTAL W, using sequences obtained from the NCBI Gene Bank. The presentation of a neighbor-joining tree, which was further tested by bootstrap analysis with 1,000 replicates using MEGA 11.0 software, was performed to identify the LAB isolates.

### Safety analysis of LAB

#### Determination of antibiotic susceptibility

The susceptibility of the LAB to antibiotics was tested as reported previously (Wang et al., 2021). All the antibiotics were purchased from Carl Roth, Karlsruhe, Germany. The LAB were tested against 30 μg kanamycin (Kan), 25 μg streptomycin (Str), 10 μg gentamicin (Gen), 30 μg vancomycin (Van), 15 μg erythromycin (Ery), 30 μg chloramphenicol (Chl), 30 μg tetracycline (Tet), 2 μg clindamycin (Cli), 10 μg ampicillin (Amp), and 10 μg penicillin (Pen) using the disc diffusion method. The concentration of antibiotics was selected according to the EFSA guidelines (Aquilina et al., 2012). The agar plates were examined for the presence or absence of zones of inhibitions after incubation at 37°C for 24 h.

### Hemolytic ability test

Hemolytic activity was performed as described in a previous study (Zhang et al., 2022). Overnight cultures of selected LAB were streaked on 5% defibrinated sheep blood agar plates and incubated at 37°C for 48 h. After incubation, the plates were observed for α-hemolysis (dark and greenish zones), β-hemolysis (lightened –yellow or transparent zones), and γ-hemolysis (no change or no zones).

### Mucin degradation test

The mucin degradation ability of the LAB was assessed using a previously reported method with slight modification (Daliri et al., 2022) Briefly, the LAB strains were grown in MRS broth supplemented with both 0.5% (w/v) glucose and 0.5% (w/v) mucin. After inoculation, the cultures were incubated at 37°C for 48 h under aerobic conditions. The bacterial growth was estimated every 6 h by measuring absorbance at 600 nm. *E. coli* ATCC 25922 was used as positive control and grown in Tryptic soy broth (Carl Roth, Karlsruhe, Germany) containing 0.5% (w/v) glucose supplemented with or without 0.5% (w/v) mucin (Sigma- Aldrich, Poznań, Poland) and cultured at 37°C for 48 h under aerobic conditions. The optical densities were measured using a spectrometer (Eppendorf Bio spectrometer®, Hamburg, Germany). The initial optical density value of the media was deducted from the final value for each test sample.

### Gelatin degradation test

The gelatin degradation ability of the LAB was investigated using MRS media containing 3% (w/v) gelatin (Sigma- Aldrich, Poznań, Poland) according to the method reported by Daliri et al. (2022). *Staphylococcus aureus* ATCC 6538 was used as a reference for quality control and was grown on tryptone soya broth (TSB) containing 3% (w/v) gelatin. Gelatin degrading ability was evaluated by the presence of a clear zone around the bacteria colony.

### Search for antimicrobial resistance genes, virulence factors, and plasmid

The bacteria genomes were screened against two antimicrobial resistance gene databases: the ResFinder server 4.1 (https://cge.food.dtu.dk/services/ResFinder/ accessed on 27.02.2023) and ResFinderFG 2.0 server (https://cge.food.dtu.dk/services/ResFinderFG/ accessed on 27.02.2023). Search for virulent factors was performed using the VirulenceFinder-2.0 server (https://cge.food.dtu.dk/cgi-bin/webface.fcgi?jobid=63FCC7FC00005B2B4068D7E0;wait= assessed on 27.02.2023). Plasmids were searched from the genome data by screening the contigs against the PlasmidFinder server 2.1 (https://cge.food.dtu.dk/services/PlasmidFinder/ assessed on 27.02.2023).

### Statistical analysis

All the experiments were performed in triplicate. Results were statistically analyzed by one-way ANOVA and two-way ANOVA and expressed as mean ± standard deviation calculated at a 95% confidence level. Tukey’s test was employed to examine differences between means at *p* < 0.05. All statistical analyses were performed using GraphPad Prism version 8.4.3 (GraphPad Software Inc., Boston, United States).

## Results

### Isolation of LAB

In this study, 23 pure bacterial colonies were obtained from various fermented food samples from Hales Turgus Market (Vilnius, Lithuania). These colonies were isolated from fermented pear (6 isolates), fermented cherry tomato (5 isolates), fermented Lithuanian cucumber, (6 isolates), and fermented orange (6 isolates)(Supplementary Table S1). These isolates were stored in the probiotic library provided by the Department of Microbiology, Faculty of Life Sciences Centre, Vilnius University, Vilnius, Lithuania.

### Screening of probiotic properties of isolated bacteria

#### Resistance to Low pH

**Figure 1:**
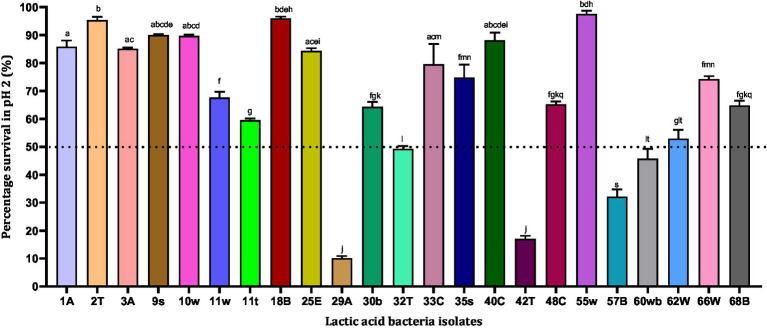
Acid resistance of lactic acid bacteria in phosphate-saline buffer (pH 2). Values are expressed as mean ± standard deviation (*n* = 3). Bars with the same lower-case letters are not significantly different, whereas those with different lower-case letters are significantly different (*p* < 0.05). The dotted line represents the minimum percentage survival requirement of the individual isolates.

During the process of digestion, the stomach lining produces gastric acid with a pH between 1 and 3, which is very acidic. This low pH plays a key role in the digestion of proteins by activating digestive enzymes, which together break down the long chains of amino acids of proteins. Hence in our study, we subjected the strains to pH 2 to observe their survival ability in *in vitro* conditions. Out of 23 isolates, only 18 strains showed survival abilities >50%. Among the strains that were tested, 55w, 18B, 2 T, and 9 s showed the highest survival abilities of 97.58, 96.02, 95.37, and 90.03%, respectively. The strains showing <50% survival abilities (LAB 57B, 42 T, and 29A) were excluded from further analysis since the drastic reduction in their survival ability could indicate fewer chances of surviving further harsh gastrointestinal conditions (Figure 1). However, the 18 strains showing resistance to low pH were tested for their tolerance to pepsin.

### Tolerance to pepsin

**Figure 2:**
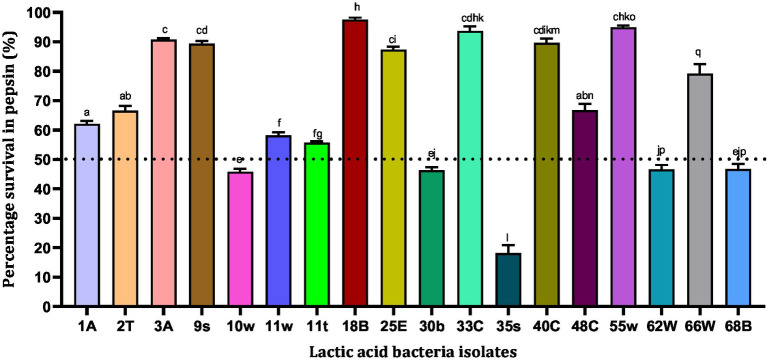
Resistance of lactic acid bacteria to pepsin (pH = 2). Values are expressed as mean ± standard deviation (*n* = 3). Bars with the same lower-case letters are not significant, whereas those with different lower-case letters are significantly different (*p* < 0.05). The dotted line represents the minimal requirement of survival of the individual isolates.

Pepsin is a digestive enzyme that breaks down proteins into smaller peptides. It is produced in the stomach lining and is one of the main digestive enzymes present in the digestive systems of humans and many other animals. However, pepsin is activated at a low pH, and we tested the survival ability of isolates in the presence of pepsin at pH 2. In all, 18 LAB isolates were tested for their tolerance to pepsin. According to our results, four bacterial isolates showed pepsin resistance above 90% in the order 18B (97.58%) > 55w (94.99%) > 33E (93.74%) > 3A (90.75%), while 35 s (18.11%) showed the least survival ability (< 20%) (Figure 2). The least resistance to pepsin was observed by five strains, 35 s (18.11%) < 10w (45.83%) <30b (46.35%) <62 W (46.63%) < 68B (46.77%), with the least survival abilities (<50%) being therefore excluded from subsequent experiments.

### Resistance to bile salts and pancreatin

**Figure 3:**
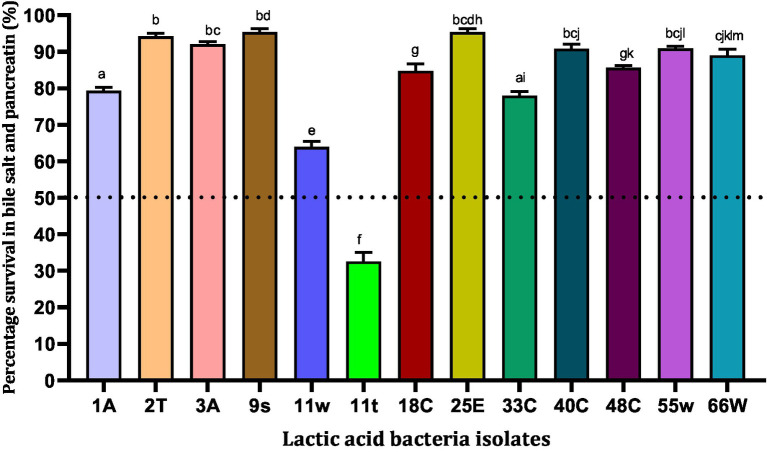
Viability of lactic acid bacteria in the presence of simulated intestinal fluid (0.3% (w/v) bile salt and 1 mg/mL pancreatin in peptone water, pH 7.2). Values are expressed in mean ± standard deviation (*n* = 3). Bars with the same lower-case letters are not significantly different, whereas those with different lower-case letters are significantly different (*p* < 0.05). The dotted line represents the minimal requirement of survival of the individual isolates.

Bile salts are steroid acids produced in the liver and stored in the gallbladder that help in the digestion of fats. The Pancreatin enzyme is produced by the pancreas and is important for digesting fats, proteins, and sugars. In this study, 13 LAB isolates showed varying levels of resistance to bile salts and 1 mg/mL pancreatin after 6 h of exposure (Figure 3). A total of six LAB strains, 25E (95.40%) > 9 s (95.37%) > 2 T (94.20%) > 3A (92.07%) > 55w (90.90%) > 40C (90.83%), were found to be highly tolerant (>90%) to simulated intestinal fluid after 6 h of incubation (Figure 3). Strains 9 s and 25E showed high tolerance to bile salts and pancreatin and their percentage rates of survival were 95.37 and 95.40%, respectively. Meanwhile, strain 11 t showed the lowest survival ability of 32.60%. In all, 12 strains showed at least 50% resistance against bile salts and pancreatin (Figure 3). Hence, these 12 strains were subjected to functional characterization.

### Functional characterization of selected probiotic candidates

#### Antimicrobial activity

Probiotics produce metabolites that can be useful for the host. Bacteriocin is a metabolite that hinders and/or suppresses the growth of pathogenic bacteria in the gut. To investigate their antimicrobial property, five pathogenic bacteria (*S. aureus* ATCC2913, *S. typhimurium* ATCC 14028, *S. pyogens* ATCC 12384, *S. pyogens* ATCC 19615, and *K. pneumoniae* ATCC 13883) were treated with the CFS of the selected LAB CFS. Only CFS from strains 18B, 25E, 48C, and 66 W inhibited these pathogens at varying degrees (Table 1). Strains 2 t, 11w, and 33E inhibited the growth of only two pathogenic microorganisms. Isolate 18B, 25E, and 48C hindered the growth of at least four pathogenic microorganisms, whereas 66 W showed an antagonistic ability to all the pathogens tested. Therefore, all 12 strains were subjected to further studies.

**Table 1:** The inhibitory ability of probiotic candidates against various pathogenic bacteria.

Culture	*Staphylococcus aureus* ATCC 29213	*Salmonella Typhimurium* ATCC 14028	*Streptococcus pyogenes* ATCC 12384	*Streptococcus pyogenes* ATCC 19615	*Klebsiella pneumoniae* ATCC 13883
1A	10.75 ± 0.5	12.00 ± 0.81	–	15.25 ± 0.56	–
2 T	–	13.75 ± 0.5	–	–	18.00 ± 0.81
3A	12.75 ± 0.5	13.25 ± 0.5	12.75 ± 0.5	–	–
9 s	11.75 ± 0.5	12.75 ± 0.5	11.75 ± 0.5	–	–
11w	14.25 ± 0.5	–	–	–	16.5 ± 1
18B	14.25 ± 0.5	–	15.62 ± 0.75	17.25 ± 1.25	18.75 ± 1.5
25E	16.75 ± 0.5	14.25 ± 0.5	15.25 ± 0.5	17.5 ± 0.57	17.5 ± 0.57
33E	–	–	–	12.75 ± 0.5	18.5 ± 1.29
40C	11.5 ± 0.57	11.5 ± 0.57	–	–	–
48C	14.75 ± 0.5	15.25 ± 0.5	–	18.00 ± 0.81	19.5 ± 0.57
55w	–	15.25 ± 0.5	12.25 ± 0.5	–	18.5 ± 0.57
66 W	11.25 ± 0.5	14.5 ± 0.057	11.25 ± 0.5	13.25 ± 0.95	19.5 ± 0.57

Results are expressed as the zone of inhibition in mm. The results are expressed as the means ± standard deviations of three independent replicates (*n* = 3). (−) indicates no zone of inhibition.

### Trolox equivalent antioxidant concentration

**Figure 4:**
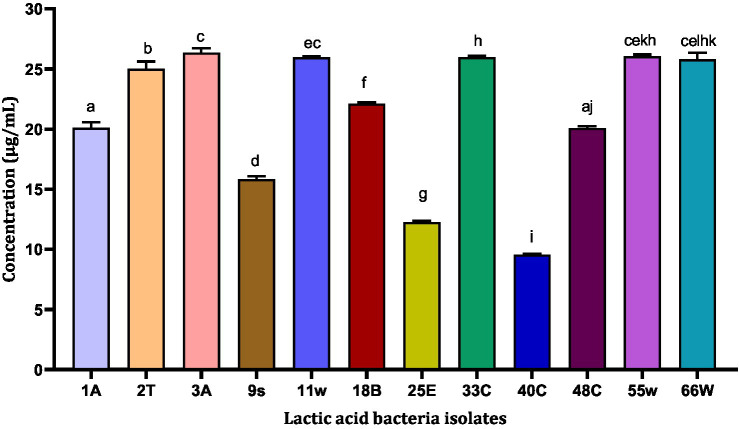
Trolox equivalent antioxidant concentration of 13 LAB CFS. Results are expressed as mean of triplicate values ± standard deviation (*n* = 3). Bars with the same lower-case letters are not significantly different, whereas those with different lower-case letters are significantly different (*p* < 0.05).

The antioxidant property of the probiotic bacteria can have an important role in anti-aging functions and in scavenging free radicals from the body. For this reason, TEAC values were evaluated for LAB CFS. The highest TEAC was exhibited by isolates 3A and 55w with TEAC of 26.37 μg/mL and 26.06 μg/mL, respectively. The least TEAC was shown by isolate 40C with a TEAC of 9.57 μg/mL (Figure 4). Even though these isolates showed different antioxidant abilities, they were all tested for their potential gut colonization abilities.

### Bacterial colonization ability

**Figure 5:**
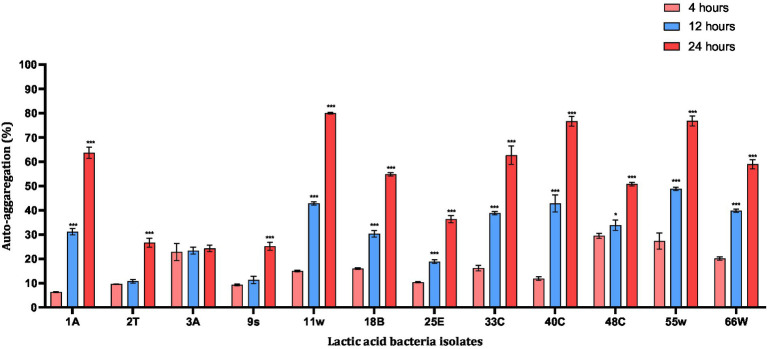
Auto-aggregation abilities of probiotic candidates after 20 h incubation at 37°C. Each value represents the mean ± standard deviation of three independent readings (*n* = 3). ^*^Significant differences at *p* < 0.05, and ^***^significant difference at *p* < 0.001.

Auto-aggregation of probiotic strains appeared to be necessary for adhesion to intestinal epithelial cells. Among the LAB tested, 11w showed the highest auto-aggregation ability of 79.96% after 24 h. Compared to all the probiotic candidates 11w, 40C, and 55w showed aggregation of 79.96, 76.63, and 76.76%, respectively, after 24 h, whereas 3A (24.26%) showed the least <25% after 24 h. Four strains, 2 T, 3A, 9 s, and 25E, showed the least aggregative potential of 26.63, 24.26, 25.18, and 36.37% (<50%), respectively, after 24 h and were excluded. Eight strains that exhibited an aggregative property of over 50% were further evaluated for their adhesion ability on HCT116 cells (Figure 5).

### Bacterial adhesion to HCT116 cells

**Figure 6:**
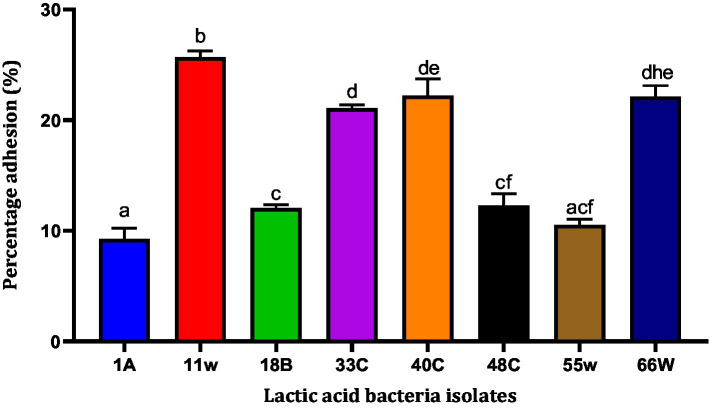
The ability of LAB to adhere to the HCT-116 cell lines. Results are expressed as mean of triplicate values ± standard deviation (*n* = 3). Bars with the same lower-case letters are not significant, whereas those with different lower-case letters are significantly different (*p* < 0.05).

The HCT116 cell model (a human-cloned colon adenocarcinoma cell) structure and function are very similar to those of the highly differentiated intestinal epithelial cells. They possess the same microvilli, tight connection, and cell polarity, which can be utilized to simulate the function of intestinal epithelial cells *in vitro*. Therefore, we chose this cell model to identify the adhesion characteristics of LAB. Nine LAB isolates adhered to HCT116 cells. Adhesion levels of these LAB isolates to HCT116 cells varied from <10 to >25%. LAB 11w showed the best adhesion ability of 25.671 ± 0.43% compared to isolate 1A, which had the least adhesion ability of 9.26 ± 0.97% (Figure 6). Among the eight isolates, 1A, 18B, and 55w showed adhesion percentages <12%. For this reason, they were excluded from further studies.

### Influence on growth in the presence of galactooligosaccharides

**Figure 7:**
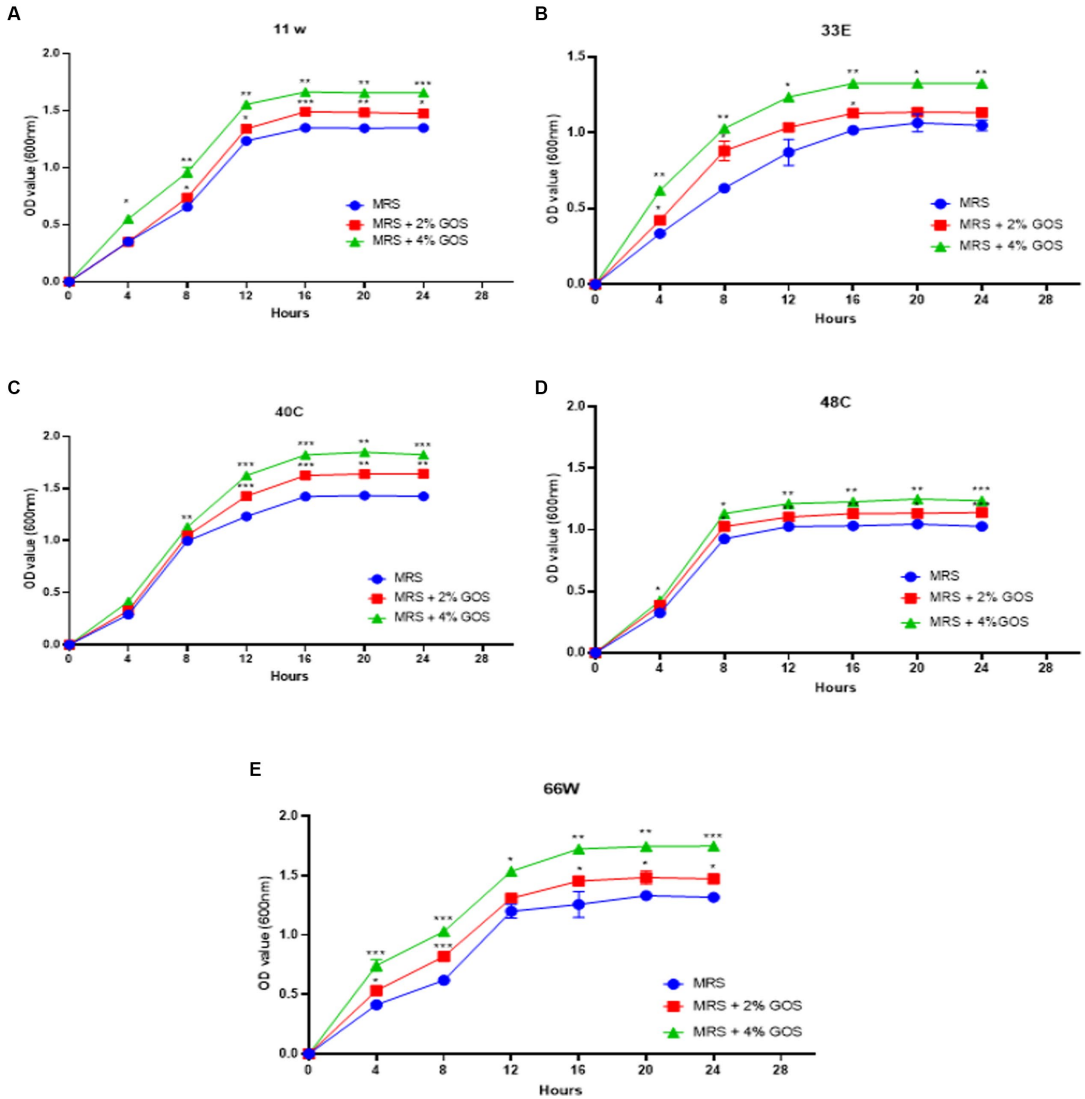
The growth curves of LAB isolates were measured at 600 nm with 2% GOS and 4% GOS. Growth curve of **(A)** 11w supplemented with 2% GOS compared with 11w with 4% GOS, **(B)** growth curve of 33E supplemented with 2% GOS compared with 33E 4% GOS, **(C)** growth curve of 40C supplemented with 2% GOS compared with 40C 4% GOS, **(D)** growth curve of 48C supplemented with 2% GOS compared with 4% GOS, and **(E)** the growth curve of 66 W supplemented with 2% GOS compared with 66 W 4% GOS. Each value represents the mean ± standard deviation of three independent readings (*n* = 3 ± SD). ^*^Significant differences at *p* < 0.05, ^**^significant difference at *p* < 0.01, and ^***^significant difference at *p* < 0.001.

Prebiotic administration increases the growth of beneficial bacteria and promotes the growth of probiotics. GOS is a very well-known prebiotic that has been employed in therapeutic uses and administered with probiotics to improve the health of animals/humans. In our study, all the LAB strains showed significant growth in the presence of GOS. Increased growth was observed in a concentration-dependent manner. The OD values of each isolate differ from others, indicating that growth is strain specific. Isolates 11 w, 33E, 40C, 48C, and 66 W showed an increase in growth when supplemented with 2% GOS, and their growth escalated after the concentration was increased to 4% (Figure 7).

### Tryptophan production by LAB

Tryptophan is an essential amino acid that cannot be produced by humans but is produced by bacteria harboring in the gut. This metabolite is known to influence the psychology of mammals by inducing quality sleep, mood enhancement, strengthening pain tolerance, and having anti-depression and anti-anxiety effects. The bacteria were tested in the presence of glucose and 4% GOS. LAB isolate 11w produced 16.63 ± 2.25 μM of tryptophan and 40C produced 2.64 ± 0.5 μM when supplemented with 4% GOS, when compared to glucose in their media in 4% GOS supplementation (Table 2).

**Table 2:** Tryptophan production by LAB CFS by HPLC-MS in 4% GOS.

LAB isolates	TSB + Glucose (μM)	TSB + 4% GOS(μM)
11w	9.95 ± 0.24	16.63 ± 2.25
33E	ND	ND
40C	ND	2.64 ± 0.56
48C	ND	ND
66 W	ND	ND

Values are expressed in mean ± standard deviation (*n* = 2). ND indicates no tryptophan production.

### Molecular identification of probiotic candidates

**Figure 8:**
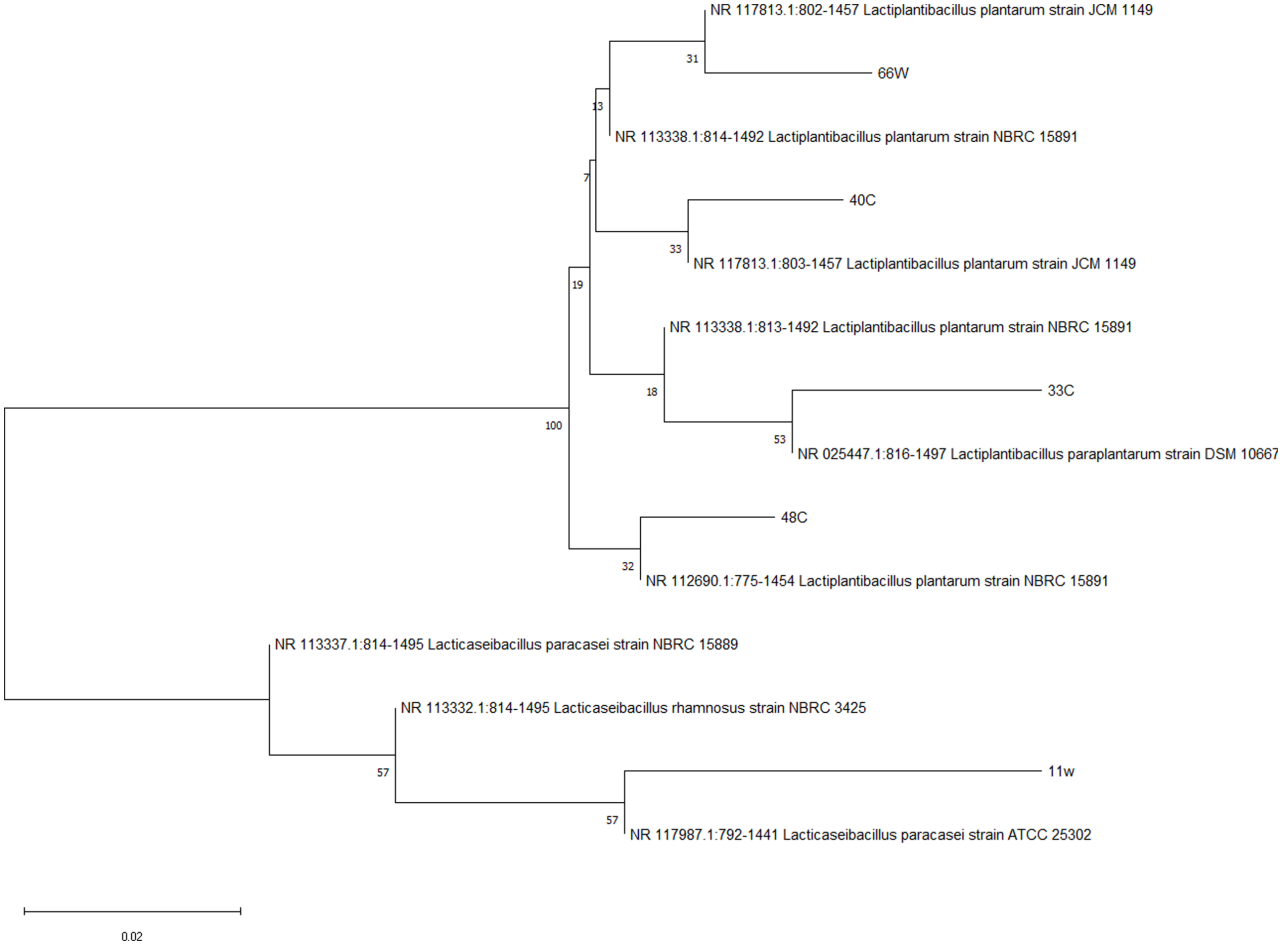
Neighbor-joining phylogenetic tree of LAB isolates based on 16S rRNA gene sequences. Bootstrap values based on 1,000 replications are listed as percentages at branch points. The scale bar represents a 0.02% divergence.

The identification of LABs was done by 16S RNA sequencing and later confirmed by whole genome sequencing. Table 3 presents the identification and accession number belonging to the closest neighbor of the tested isolates. These isolates, which showed the closest match to the reference sequence in the NCBI GeneBank, were identified as *Lacticaseibacillus paracasei* (11w), *Lactiplantibacillus paraplantarum* (33E), *Lactiplantibacillus plantarum* (40C), *Lactiplantibacillus plantarum* (48C), and *Lactiplantibacillus paraplantarum* (66 W) (Figure 8).

**Table 3:** Species identification of LAB isolates by 16S rRNA sequencing.

Arbitrary name	Strain	Source	Closest Homolog	Similarity (%)	Closest homolog Gene Bank Accession Number (NCBI)
11w	*Lacticaseibacillus paracasei* 11w	Fermented pear	*Lacticaseibacillus paracasei* strain R094	99.71%	NR_025880.1
33	*Lactiplantibacillus paraplantarum* 33E	Fermented cherry tomato	*Lactiplantibacillus paraplantarum* strain DSM 10667	99.56%	NR_025447.1
40C	*Lactiplantibacillus plantarum* 40C	Fermented cherry tomato	*Lactiplantibacillus plantarum* strain JCM1149	99.56%	NR_117813.1
48C	*Lactiplantibacillus plantarum* 48C	Fermented cherry tomato	*Lactiplantibacillus plantarum* strain CIP 103151	99.71%	NR_104573.1
66 W	*Lactiplantibacillus paraplantarum* 66 W	Fermented cucumber	*Lactiplantibacillus paraplantarum* strain DSM 10667	99.85%	NR_025447.1

### Safety analysis of probiotic candidates

#### Antibiotic susceptibility of probiotic candidates

All six LAB tested in this study were resistant to vancomycin, streptomycin, and kanamycin, whereas they were susceptible to chloramphenicol except for isolate 48C (Table 4). Most of the strains showed resistance to gentamycin except for isolate 48C (*L. plantarum* 48C). Of the eight antibiotics tested on *L. plantarum* 48C, it showed resistance to only three antibiotics and was susceptible to five. Due to its poor resistance to antibiotics, we did not include it in the subsequent safety steps.

**Table 4:** Susceptibility of LAB isolates to eight antibiotics (R, Resistant; S, Susceptible).

Cultures	Van	Strep	Kana	Gent	Novo	Ampi	Erth	Chlora
11w	R	R	R	R	S	S	S	S
33E	R	R	R	R	R	S	R	S
40C	R	R	R	R	R	S	R	R
48C	R	R	R	S	S	S	S	S
66 W	R	R	R	R	S	R	S	S

Van, vancomycin; Strep, streptomycin; Kana, kanamycin; Gent, gentamycin; Novo, Novobiocin; Ampi, Ampicillin; Erth, Erythromycin; Chlora, chloramphenicol.

### Mucin degradation

**Figure 9:**
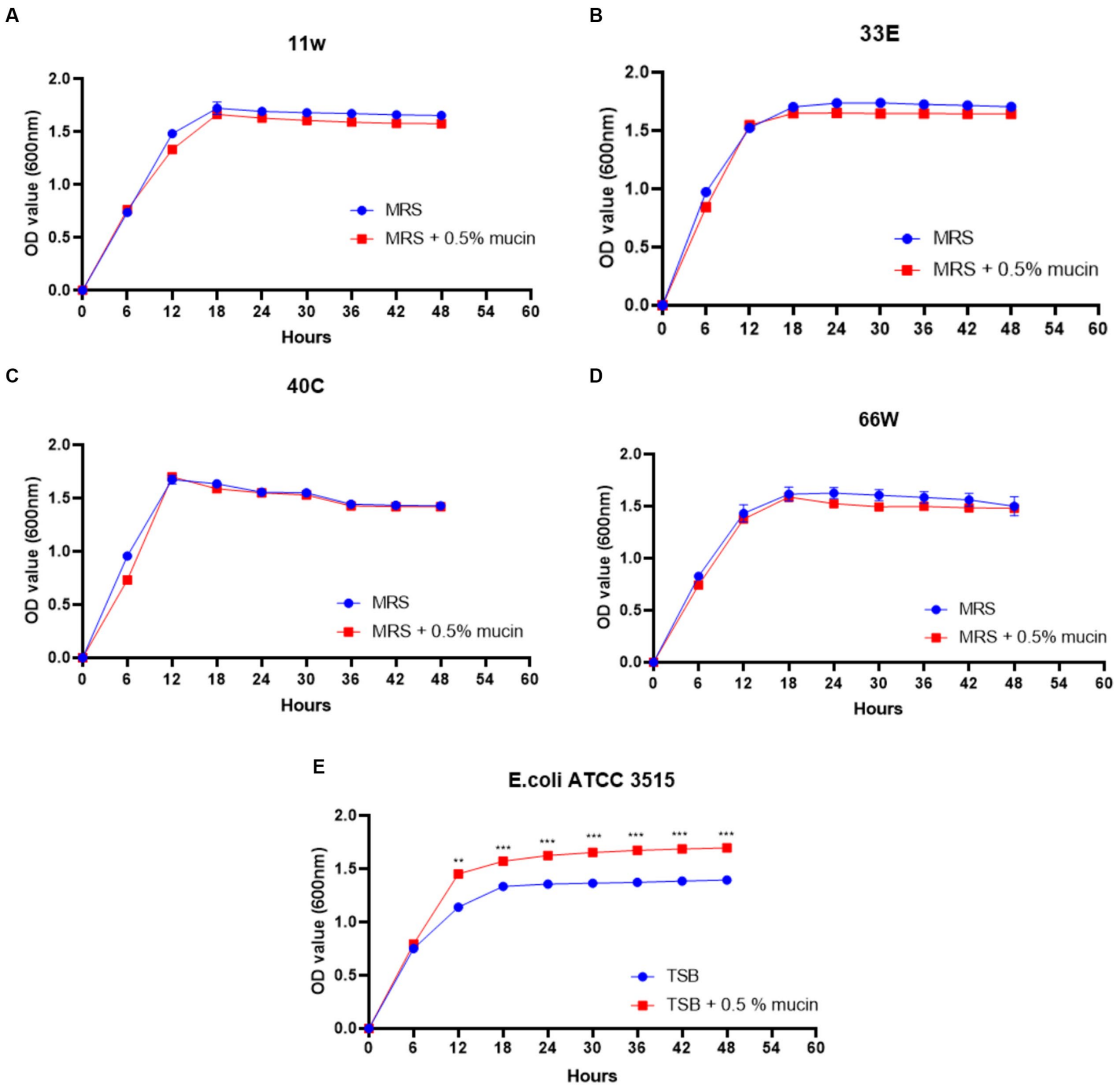
Mucin degradation ability of probiotic candidates. **(A)** growth curve of 11w (*Lacticaseibacillus paracasei* 11w) supplemented with 0.5% mucin compared with 11w without mucin, **(B)** growth curve of 33E (*Lactiplantibacillus paraplantarum* 33E) supplemented with 0.5% mucin compared with 33E without mucin, **(C)** growth curve of 40C (*Lactiplantibacillus plantarum* 40C) supplemented with 0.5% mucin compared with 40C without mucin, and **(D)** the growth curve of 66 W (*Lactiplantibacillus paraplantarum* 66 W) supplemented with 0.5% mucin compared with 66 W without mucin, **(E)** growth curve of *E. coli* ATCC 3515 supplemented with 0.5% mucin compared with *E. coli *ATCC 3515** without mucin. ^*^Significant differences at *p* < 0.05, ^**^significant difference at *p* < 0.01, and ^***^significant difference at *p* < 0.001.

As shown in Figure 9, the addition of mucin to TSB media extended the exponential phase of *E. coli* ATCC 35150 compared to when mucin was absent. Meanwhile, mucin supplementation did not increase the growth of any of the LAB strains.

### Hemolytic activity

There was a hemolytic zone (β-hemolysis) around the *S. aureus* colony on blood agar. Two isolates, 33E (*L. paraplantarum* 33E) and 66 W (*L. paraplantarum* 66 W), showed mild hemolysis (α-hemolysis), i.e., greenish color due to surrounding the colonies. This indicates that 33E and 66 W exhibit subtle hemolysis of the erythrocytes. While the colonies of two isolates, 11w and 40C, had no zone effect (γ-hemolysis), indicating that they had no hemolytic activity. Only 11w and 40C proceeded to the subsequent safety steps of the study.

### Gelatin hydrolysis

**Figure 10:**
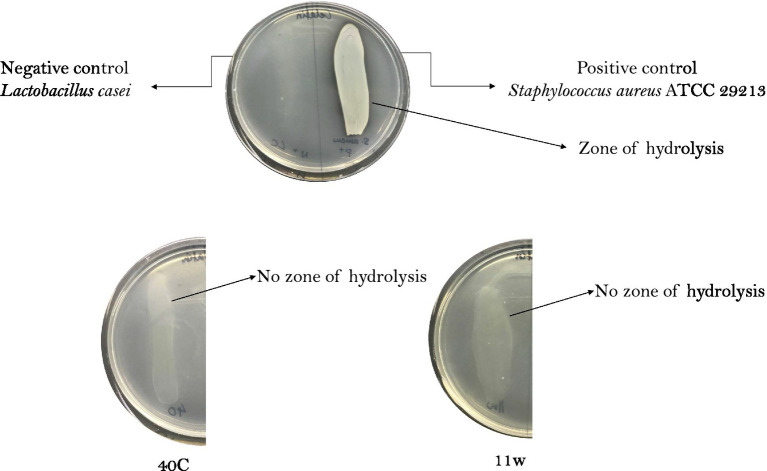
Gelatin degradation ability of LAB. Bacteria with gelatinase activity exhibited by positive control; *Staphylococcus aureus* ATCC 29213 shows clear zones around the bacteria colony, whereas the negative control *Lactobacillus casei* has no zone formation. 11w = *Lacticaseibacillus paracasei* 11w, and 40C = *Lactiplantibacillus plantarum* 40C. *Staphylococcus aureus* ATCC 6538 was used as a positive control. (+) indicates positive results and (−) indicates negative results.

Gelatin is a natural biomacromolecule derived from collagen in animal skin, bones, and connective tissues. Gelatinase is an enzyme produced by several bacteria that is capable of degrading gelatin. Figure 10 shows that none of the LAB isolates degraded gelatin. Meanwhile, only *S. aureus* ATCC 29213 (positive control) showed gelatin degrading ability, as it showed a clear zone of inhibition around the colony. Three isolates did not have the property of breaking down gelatin, thus, they were considered safe.

### Investigation for plasmids in LAB

Plasmids can be transferred from one bacterium to another through the conjugation process. The antibiotic resistance of one bacterium can be acquired by pathogenic bacteria. Therefore, for this reason, plasmids were searched from the genome data by screening the contigs against the PlasmidFinder server 2.1 (https://cge.food.dtu.dk/services/PlasmidFinder/ assessed on 27.02.2023). Isolate 11w showed intrinsic resistance to the antibiotics (Supplementary Figure S1), whereas 40C harbored plasmid (Supplementary Figure S2).

## Discussion

The human gastrointestinal tract has several enzymes and pH conditions that may inhibit microbial survival (Smith, 2003; Singh et al., 2022). Hence, probiotics must therefore survive these harsh conditions to elicit their health potential. In the current study, different probiotic LAB strains were isolated from Lithuanian fermented foods, and 23 morphologically different isolates were screened *in vitro* for survival in gastrointestinal conditions. Interestingly, after consumption, probiotics first encounter lysozymes in saliva, following through the hostile environment of the stomach and then to the intestine (Conway et al., 1987). Since the pH of gastric juice is between 1.5 and 3.5, it is necessary to test the survival ability of probiotic candidates in low-pH environments and pepsin (Ruiz-Pulido and Medina, 2021). Low pH values in the gastric juice impair the cell membrane and the cell wall of the bacteria; influencing the membrane pathway, leading to undesirable metabolic processes, energy depletion, and finally cell death (Sengupta et al., 2013). Survival under acid conditions is executed by adapting to low pH through a mechanism called acid tolerance response (Wendel, 2022). In this study, *L. paracasei* 11w showed a strong survival rate of 67.61% in low pH and this is similar to an earlier study that reported that *L. paracasei* T40 isolated from Tenate cheese has strong resistance to low pH because it has a system that automatically transports protons and lactic acid to the cell exterior (Falfán-Cortés et al., 2022). Indeed, the acid tolerance of the bacterium could be attributed to the high number of membrane-bound H + -ATPase they possess (Guan and Liu, 2020). These H + -ATPases play critical roles in maintaining intracellular pH and hence protect the cells from acid damage (Guan and Liu, 2020). The survival ability of different LAB at low pH depends on their phenotype characteristics and environmental conditions (Ko et al., 2022). The resistance to low pH of these LAB is very important as they can survive and proliferate in harsh gastrointestinal conditions.

In addition to the low pH, probiotics must survive the harsh proteolytic ability of pepsin in the stomach. In the present study, 13 out of 23 isolates showed strong tolerance towards pepsin and low pH. Earlier studies showed that LAB such as *L. paracasei* and *P. pentosaceus* are resistant to pepsin (Mantzourani et al., 2019). The ability of LAB to survive in GIT could be due to the developed mechanisms to survive in acidic conditions by producing alkaline compounds in the cell cytoplasm, altering their cell envelopes, inducing stimulation of H + -ATPase, and/ or ingestion of protons inside cells (Ayyash et al., 2021). After exiting the stomach, probiotics enter the duodenum where they encounter bile salts and pancreatin. Pancreatin is a combination of digestive enzymes that is essential for digesting fats, proteins, and sugars, whereas, bile is a digestive fluid that solubilizes lipid and lipid-soluble vitamins for digestion (Begley et al., 2005; Prete et al., 2020). A criterion for probiotic bacteria selection is the ability to tolerate the presence of pancreatic enzymes in the gut. In our study, isolate *L. plantarum* 40C showed tolerance to pancreatin and this result is in agreement with earlier studies, which demonstrated that *L. plantarum* SAM2 was resistant to pancreatin (EL-Sayed et al., 2022). A high bile acid concentration is injurious to the microbiota and studies have shown that *Lactobacillus plantarum* growth can be retarded by bile (Prete et al., 2020). In this study, however, 12 strains showed resistance to a mixture of bile salts and pancreatin after 6 h of exposure (Figure 3). Different LAB have particular genes, such as the *ula*A *and ula*B genes (found in *L. plantarum* S83), which help them to tolerate different bile salt conditions. However, resistance to bile salts is strain-specific (Ruiz et al., 2013).

The antibacterial property of LAB is complex and multifarious, and they do this mainly by exhibiting antagonism against pathogen growth and binding (Hu et al., 2019). It was observed that LAB 25E showed a strong inhibition against *Staphylococcus aureus* and *Streptococcus pyogenes* (Gram-positive bacteria), as well as *S. typhimurium* and *K. pneumoniae* ATCC 13883 (Gram-negative bacteria). It has been suggested that the inhibitory effects of potential probiotic strains against Gram-positive pathogenic bacteria are more promising than Gram-negative pathogenic bacteria (Shahverdi et al., 2023). Pathogen inhibition by probiotics is usually done via the production of antimicrobial compounds (Śliżewska et al., 2021) such as organic acids (mainly acetic acid and lactic acid), hydrogen peroxide, and/ or bacteriocin (Zare and Lashani, 2018). Furthermore, the suppression of pathogenic bacteria by LAB can also be influenced by numerous chemical, physical, and nutritional environmental factors (Hung et al., 2021). Our findings are similar to those of previous studies that showed that CFS of *L. plantatrum* (Leslie et al., 2021) had antimicrobial activity against Gram-positive and Gram-negative bacteria. Similarly, numerous studies propose health benefits postulated by the intake of viable LAB strains, which were correlated with their antimicrobial potential such as modulation of microbiota, suppression and prevention of pathogens (Liu et al., 2021), and immune modulation of the human host (Cristofori et al., 2021).

TEAC helps to evaluate the antioxidant capacity of food, beverage, and nutritional supplements using Trolox as standard (Maksimović and Dragišić Maksimović, 2017; Xiao et al., 2020). In a recent study on corn milk fermented by 20 isolates of *Limosilactobacillus fermentum*, *L. fermentum* L15 exhibited the strongest TEAC of 0.348 ± 0.005 mmol/L (Xu et al., 2022). In this study, the CFS of the LAB samples were analyzed, and the results of the TEAC confirm the free radical scavenging ability of each isolate (Figure 4). Two isolates, 3A and 55w, had the strongest antioxidant capacities of 26.37 μg/mL and 26.06 μg/mL, respectively, which indicates that the LAB released antioxidant compounds in their supernatants during growth (Lu et al., 2021).

The ability of LAB to auto-aggregate and adhere to the colon has been reported as a good indicator of gut colonization (Benítez-Cabello et al., 2019; Zawistowska-Rojek et al., 2022). The auto-aggregation capacity of LAB has been associated with their ability to adhere to epithelial cells (Botta et al., 2014). LAB aggregation can form a barrier to exclude pathogenic strains from adhering to the GIT (Klopper et al., 2018). In this study, the auto-aggregation of the LAB isolates increased with fermentation time (Figure 5). This was similar to reports from other studies where *L. plantarum* CCMA 0743 and *L. paracasei* CCMA 0504 showed, respectively, 38.62% ± 2.56 and 45.36% ± 6.30 aggregation after 5 h of incubation (Fonseca et al., 2021). Further analysis using HCT116 cells showed that *Lacticaseibacillus paracasei* 11w had the strongest attachment ability relative to the other strains (Figure 6). In agreement with a previous study, our results showed that the ability of LAB strains to adhere to the HCT116 cell line was strain-specific and varied even within the same species (Du et al., 2022).

Earlier studies have shown that prebiotics affect the growth and metabolism of probiotics (Slizewska and Chlebicz-Wójcik, 2020), and the growth may vary depending on the concentrations of the prebiotic used (Delgado-Fernández et al., 2019). In the present study, 2% and 4% GOS administration showed a proliferation in the growth of LAB isolates, which can be due to different concentration administration of prebiotics (Figure 7). An increase in LAB growth in the presence of GOS has a beneficial role in the mitigation of different diseases and influences the maturation and protection of the gut barrier as well as has an effect on the overall balance of the immune system (Manzoor et al., 2022; Megur et al., 2022). On the other hand, GOS administration has been shown to increase the production of essential amino acids, such as tryptophan and histidine, in *in vivo* studies (Purton et al., 2021; Saleh-Ghadimi et al., 2022). Tryptophan is an essential amino acid required for cellular energy, mood, immunity, and sleep regulation (Davidson et al., 2022). LAB-derived tryptophan metabolites are essential signals for maintaining gut homeostasis (Klaessens et al., 2022). In a study on *L. plantarum* ZJ316, this particular strain produced the tryptophan-derived metabolite indole-3-lactic acid, which resulted in the modulation of the gut by hindering the growth of pathogenic bacteria (Zhou et al., 2022). In the present study, *L. paracasei* 11w showed the production of tryptophan in the presence of glucose (9.95 ± 1.23 μM) as well as 4% GOS (16.63 ± 2.25 μM), and *L. plantarum* 40C showed production of 2.64 ± 0.56 μM in the presence of 4% GOS.

LAB are generally recognized as safe, but safety properties should be evaluated prior to administration (Ayivi et al., 2020). In this study, isolates that passed the screening and characterization steps were tested for their antibiotic resistance, hemolytic analysis, mucin degradation, and gelatinase activity. Susceptibility to antibiotics is an important criterion in selecting probiotic candidates. This is because of the possibility of horizontal gene transfer of antibiotic-resistant genes from probiotic candidates to pathogenic bacteria (Lastauskienė et al., 2021; Daliri et al., 2023b). In this study, all the strains were resistant to vancomycin, streptomycin, and kanamycin, and susceptible to chloramphenicol except for strain *L. plantarum* 40C, which showed resistance to chloramphenicol (Table 4). All the strains except 11w harbored plasmid pR18, which contains linA (a linomycin and ampicillin resistance gene). Meanwhile, only strain 66 showed resistance to ampicillin, whereas the other five strains were susceptible to ampicillin. It is likely that only strain 66 has an active linA gene, which might have resulted in its resistance to ampicillin. Therefore, no other antimicrobial factors were detected in the bacteria genome. The strains were likely intrinsically resistant to most of the antibiotics tested.

The GIT mucus layer serves as a barrier to prevent bacteria translocation, which could result in sepsis (Haussner et al., 2019). Due to this reason, the mucolytic potentials of the selected LAB were assessed *in-vitro* using *E. coli* ATCC 35150 (a known mucin-degrading bacterium) as a positive control. It was observed that the presence of mucin in MRS media did not improve the growth of the tested LAB, and this implies that the bacteria could not metabolize mucin as a carbon source for their growth. Meanwhile, as expected*, E. coli* ATCC 35150 grew better in the presence of mucin than in TSB media containing limited glucose (Figure 9).

Some bacteria are known to produce enzymes that break down phospholipids and cause rupture of the cell membrane of red blood cells (Lee et al., 2023). *S. aureus* is known for its hemolytic abilities (Zhang et al., 2016). In this study, two LAB isolates showed alpha hemolysis. The two LAB isolates, *L. paracasei* 11w and *L. plantarum* 40C, did not cause any lysis of the erythrocytes of sheep blood, and thus they have no hemolytic activities. This investigation corroborates well with previous findings that showed that *L. paracasei* and *L. plantarum* (Sun et al., 2022) were non-hemolytic.

Gelatinase is a Zn metalloproteinase secreted by pathogenic bacteria. Gelatinase hydrolyses gelatin (a structural protein in connective tissues) and it can effectively attack the host by digesting the protein components of tissue, so as to facilitate the spread of bacteria. In addition, bacteria that produce gelatinase have been shown to have high chances of translocation from the gut to the liver, spleen, heart, and mesenteric lymph nodes (Zhang et al., 2015). In the present study, we tested whether any of the LAB could hydrolyze gelatin. Probiotics must not cause gelatin liquefaction in the host by producing a gelatinase enzyme. In prior studies it is demonstrated that *Lacticaseibacillus paracasei* (Martín et al., 2023) and *Lactiplantibacillus plantarum* (Kwon et al., 2021) are gelatinase-free and can be proceeded as potential probiotics. In comparison to our study, two isolates *Lacticaseibacillus paracasei* 11w and *Lactiplantibacillus plantarum* 40C did not demonstrate hemolysis and had no gelatinase activity (Figure 10). Furthermore, the *L. plantarum* 40C isolate harbored plasmid in its genome, whereas *L. paracasei* 11w did not harbor any plasmid, which makes it the only safe option among all the 23 strains isolates tested from different Lithuanian fermented foods.

## Conclusion

Fermented foods are rich sources of LAB (probiotic bacteria), as demonstrated in the present study. However, it is imperative for their potential use that their functional potentials are studied as well as their safety assessments. We have demonstrated that most of the LAB strains isolated from Lithuanian fermented foods survived under simulated gastrointestinal conditions.

However, only five isolates, namely, *L. paracasei* 11w*, L. plantarum* 40C*, L. plantarum* 48C, *L. paraplantarum* 33C, and *L. plantarum* 66 W, displayed pathogenic bacteria inhibition, antioxidant potential, strong adhesion to gut epithelia, and high tryptophan production. After a safety assessment of the five isolates, only *L. paracasei* 11w met the safety requirements. Though *L. paracasei* 11w has demonstrated strong probiotic potential in this study, further studies are required to establish its health-promoting effects in animal models.

Results from the current study indicate that, though LAB isolated from fermented foods may have promising probiotic and functional potentials, they may pose some risks when consumed. They must, therefore, be subjected to strict safety assessments before use.

## Data availability statement

The datasets presented in this study can be found in online repositories. The names of the repository/repositories and accession number(s) can be found in the article/Supplementary material.

## Ethics statement

Ethical approval was not required for the studies on humans in accordance with the local legislation and institutional requirements because only commercially available established cell lines were used.

## Author contributions

AM, TB, and JS: investigation. AM, ED, and EL: management. AM and ED: formal analysis. AM, ED, and AB: supervision, validation, and writing−original draft. AM, ED, AB, TB, JS, and EL: writing−review & editing. All authors contributed to the article and approved the submitted version.

## Funding

This project has received funding from European Regional Development Fund (project No 09.3.3-LMT-K-712-23-0117) under a grant agreement with the Research Council of Lithuania (LMT LT).

## Conflict of interest

The authors declare that the research was conducted in the absence of any commercial or financial relationships that could be construed as a potential conflict of interest.

## Publisher’s note

All claims expressed in this article are solely those of the authors and do not necessarily represent those of their affiliated organizations, or those of the publisher, the editors and the reviewers. Any product that may be evaluated in this article, or claim that may be made by its manufacturer, is not guaranteed or endorsed by the publisher.

## Footnotes

^1^ www.ncbi.nlm.nih.gov

## Abbreviations

LAB: Lactic acid bacteria
GIT: Gastrointestinal tract
CFS: Cell-free culture supernatants
CFU: Colony forming units
TSB: Tryptone soya broth
GOS: Galactooligosaccharides
