# Supplementary material for: In vitro screening and characterization of lactic acid bacteria from Lithuanian fermented food with potential probiotic properties
Source: Front Microbiol. 2023 Sep 8;14:1213370. doi: 10.3389/fmicb.2023.1213370 (PMC10516296; doi:10.3389/fmicb.2023.1213370)

**Supplementary table 1.** Bacterial strains and their sources.

| Isolates | Sources |
| --- | --- |
| 1A | Fermented orange |
| 2T | Fermented orange |
| 3A | Fermented orange |
| 9s | Fermented orange |
| 10w | Fermented pear |
| 11w | Fermented pear |
| 11t | Fermented pear |
| 18B | Fermented pear |
| 25E | Fermented pear |
| 29A | Fermented pear |
| 30b | Fermented cherry tomato |
| 32T | Fermented cherry tomato |
| 33C | Fermented cherry tomato |
| 35s | Fermented orange |
| 40C | Fermented cherry tomato |
| 42T | Fermented orange |
| 48C | Fermented cherry tomato |
| 55w | Fermented cucumber |
| 57B | Fermented cucumber |
| 60wb | Fermented cucumber |
| 62W | Fermented cucumber |
| 66W | Fermented cucumber |
| 68B | Fermented cucumber |

Supplementary Figure 1. Investigation of plasmids in isolate 11w.


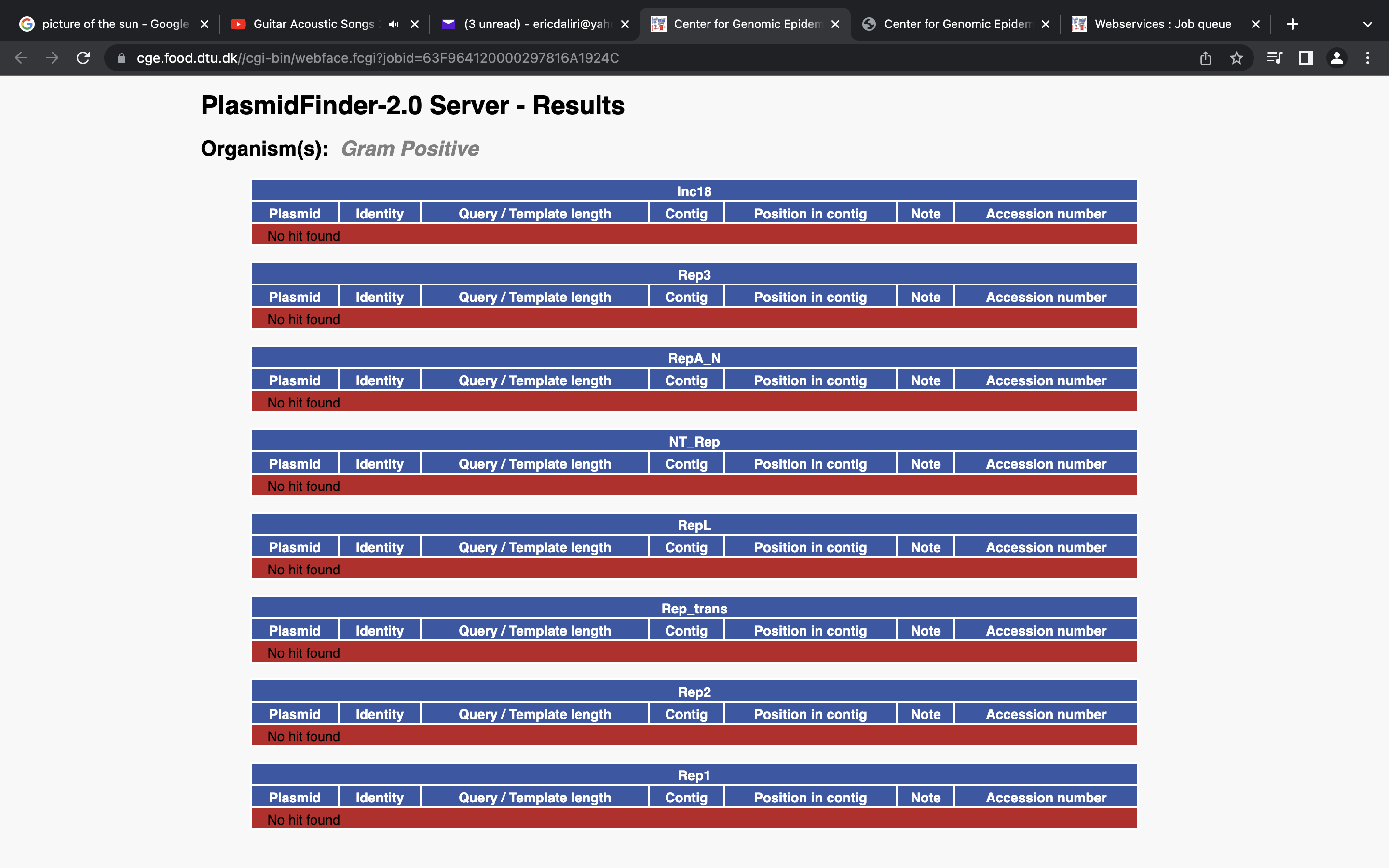


Supplementary figure 2. Investigation of plasmid in isolate 40C.


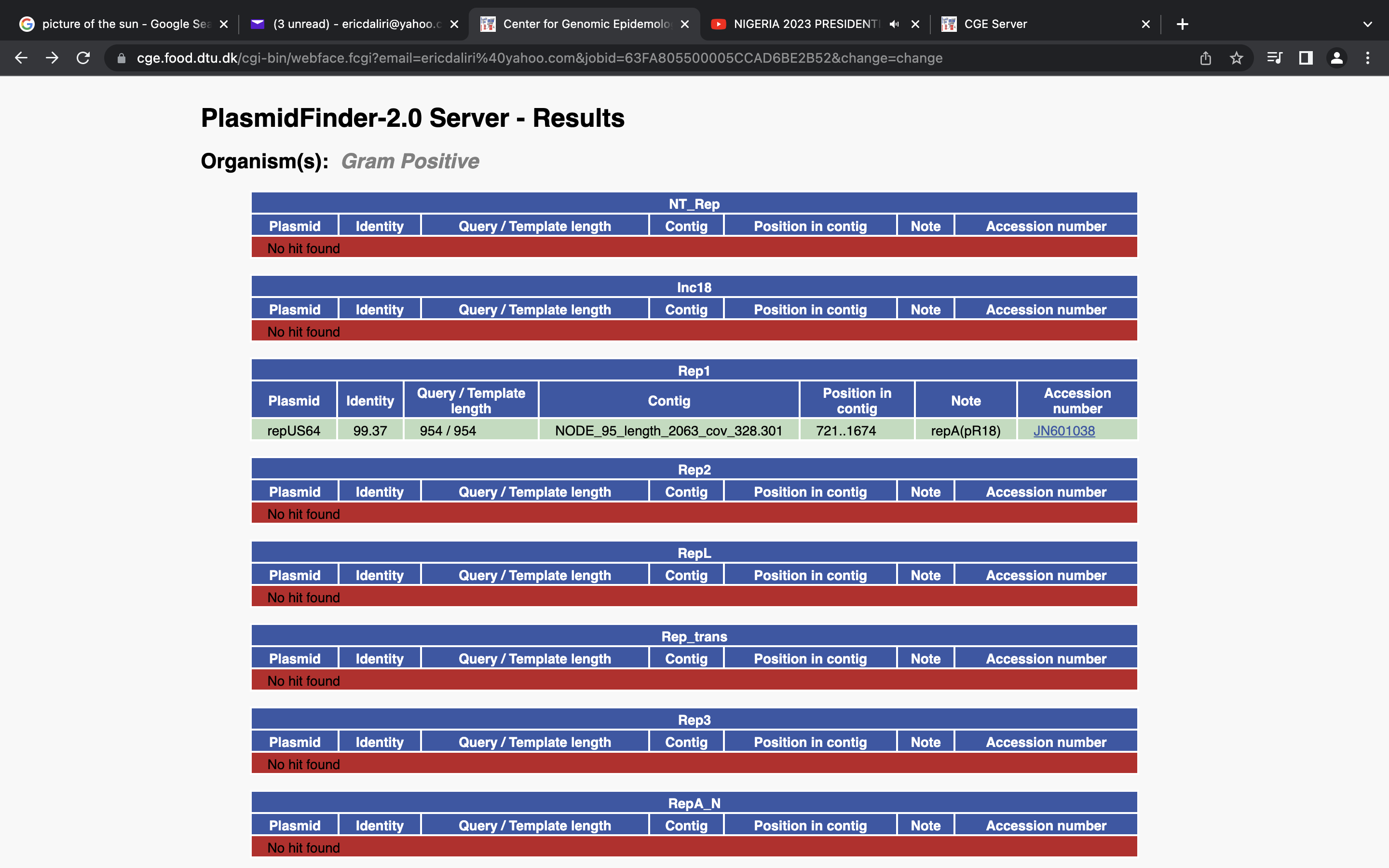

Supplement: Supplementary file 1 [file Data_Sheet_1.DOCX]
